# Automatic segmentation of the solid core and enclosed vessels in subsolid pulmonary nodules

**DOI:** 10.1038/s41598-017-19101-3

**Published:** 2018-01-12

**Authors:** Jean-Paul Charbonnier, Kaman Chung, Ernst T. Scholten, Eva M. van Rikxoort, Colin Jacobs, Nicola Sverzellati, Mario Silva, Ugo Pastorino, Bram van Ginneken, Francesco Ciompi

**Affiliations:** 1 Diagnostic Image Analysis Group, Radboud University Medical Center, Nijmegen, The Netherlands; 2 Section of Radiology, University Hospital of Parma, Parma, Italy; 3 Section of Thoracic Surgery, Fondazione IRCCS Istituto Nazionale Tumori, Milano, Italy

## Abstract

Subsolid pulmonary nodules are commonly encountered in lung cancer screening and clinical routine. Compared to other nodule types, subsolid nodules are associated with a higher malignancy probability for which the size and mass of the nodule and solid core are important indicators. However, reliably measuring these characteristics on computed tomography (CT) can be hampered by the presence of vessels encompassed by the nodule, since vessels have similar CT attenuation as solid cores. This can affect treatment decisions and patient management. We present a method based on voxel classification to automatically identify vessels and solid cores in given subsolid nodules on CT. Three experts validated our method on 170 screen-detected subsolid nodules from the Multicentric Italian Lung Disease trial. The agreement between the proposed method and the observers was substantial for vessel detection and moderate for solid core detection, which was similar to the inter-observer agreement. We found a relatively high variability in the inter-observer agreement and low method-observer agreements for delineating the borders of vessels and solid cores, illustrating the difficulty of this task. However, 92.4% of the proposed vessel and 80.6% of the proposed solid core segmentations were labeled as usable in clinical practice by the majority of experts.

## Introduction

Lung cancer screening with low-dose computed tomography (CT) is currently being implemented in the US. The main purpose of this screening program is to detect lung cancer at a stage in which it is still curable, thereby reducing lung cancer mortality as has been shown in the National Lung Screening Trial^1^. In lung cancer screening and clinical routine, a large amount of nodules is encountered for which a suitable follow-up strategy needs to be determined. For this purpose, guidelines and tools have been introduced that define a clear procedure for follow-up management, such as the Lung-RADS guidelines^2^, the Fleischner guidelines^3^, and the PanCan model^4^. In these recommendation guidelines and tools, nodule characteristics on CT, such as nodule type, size, and growth, play a vital role in choosing a suitable management for each nodule.

Identification of the type of a nodule is important since some types of nodules have a higher chance of being malignant compared to others^5,6^. Several nodule types are typically considered to categorize pulmonary nodules, including non-solid and part-solid nodules. Non-solid nodules manifest as an increased hazy attenuation in the lung (i.e. *ground-glass*) in which the vascular and bronchial margins are still visible. Part-solid nodules consist of ground-glass with an area of homogeneous soft-tissue attenuation (i.e. *solid core*) in which, unlike ground-glass, vessels are not distinguishable anymore. Non-solid and part-solid nodules are typically grouped as *subsolid* nodules and are associated with a higher malignancy probability^5,6^.

Nodule growth has additionally been identified as an important CT characteristic that is associated with malignancy^2^. A previous study by de Hoop *et al*.^7^ showed that an increasing nodule *mass* in subsolid nodules is subject to less variability than volume and diameter measurements for estimating growth. Nodule mass depends on both nodule volume, which is related to the segmentation of a nodule in the 3D scan, and nodule density, which is related to the CT attenuation in Hounsfield Units (HU). Once a nodule is segmented, the nodule mass can be calculated by multiplying the mean nodule density with the nodule volume^8^.

In this study, we specifically focused on subsolid nodules consisting of a ground-glass part and potentially a solid core. Several studies have described algorithms for subsolid nodule segmentation (such as the work by Jacobs *et al*.^9^ or Messay *et al*.^10^), however only a few publications considered solid core segmentation in subsolid nodules^11,12^. Given the segmentation of the nodule, a solid core can be distinguished from ground-glass based on the HU attenuation. However, when vessels are enclosed in the subsolid nodule, a solid core and a vessel can be difficult to separate since both have a soft-tissue attenuation. Techniques based on thresholding such as the ones presented by Jacobs *et al*.^11^ or Scholten *et al*.^12^ are therefore less suitable to discriminate solid cores from vessels. Furthermore, these methods are semi-automatic as they require a manually indicated seed point.

**Figure 1:**
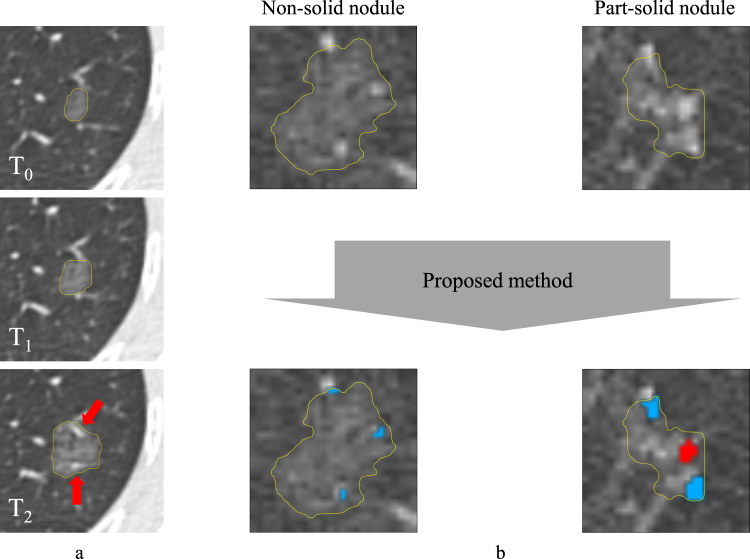
Examples of subsolid nodules that enclose vessels. (**a**) Three time points of a growing non-solid nodule. At *T*_2_ the non-solid nodule encloses two vessels (indicated by the red arrows) which, if not removed, alter the size and mass of the nodule. Furthermore, these vessels may be mistaken for a solid core which changes the type of the nodule and possibly the patient management. (**b**) An example of a non-solid and a part-solid nodule and the results of the proposed method. In this example, the detected solid core is shown in red and the detected vessels are shown in blue.

Including vessels as part of a nodule can alter nodule characteristics such as the nodule type, mass, and the size of the nodule or solid core. This can affect treatment decisions and can hamper the correlation between nodule mass and nodule growth as presented in de Hoop *et al*.^7^, making it an unreliable parameter to use in a screening setting. An example of a growing non-solid nodule is shown in Fig. 1a. Over time, this nodule encloses surrounding vessels which, if not considered, can result in an overestimated growth that changes patient management.

The goal of this paper is to automatically identify and segment vessels and solid cores in a given subsolid nodule. We formulate our method as a voxel classification problem, in which a classifier labels all voxels within a given nodule segmentation as belonging to one of the following three classes: *vessel, solid core*, or *ground-glass*. Each voxel is characterized by features related to the intensity and shape of the 3D structure it belongs to. In addition, vessels outside of the nodule are used to ensure an anatomically plausible vessel segmentation inside of the nodule. An example of a non-solid and a part-solid nodule and the results of our method is shown in Fig. 1b. We validated the proposed framework on a data set consisting of all screen-detected subsolid nodules at baseline from the Multicentric Italian Lung Disease (MILD) trial^13^. An observer study was designed involving three human experts to evaluate the results of the proposed automatic method, providing both a quantitative and a qualitative evaluation. As an additional contribution, we assessed the inter-observer variability among experts for the detection and voxel-wise delineation of vessels and solid cores in subsolid nodules.

## Results

Three experiments were performed to evaluate the proposed method. The first paragraph of this section describes the data that was used for the evaluation. Based on this data, we first evaluated whether the proposed method is able to detect vessels and solid cores within a subsolid nodule. Next, we performed a voxel-wise quantitative evaluation in which the automatically extracted vessel and solid core segmentations were compared to manually segmented reference standards. As a final evaluation, the quality of the entire vessel and solid core segmentations was assessed.

### Evaluation Data

The evaluation of the proposed method was done using all baseline low-dose CT scans (120 kV, 30 mAs) from the Multicentric Italian Lung Detection (MILD) study^13^. The study was approved by the Institutional review board of Fondazione IRCCS Istituto Nazionale Tumori di Milano, and the written informed consent was waived for the retrospective examination of the analyzed data. All CT scans were acquired with a collimation of 0.75 mm, rotation time of 0.5 s, and a pitch of 1.5. The average in-plane resolution was 0.74 mm ± 0.06 mm with one-millimeter-thick sections and a 1 mm increment. All scans were reconstructed with a sharp reconstruction kernel (Siemens B50 kernel, Siemens Medical Solutions). We selected all baseline subsolid nodules which resulted in a total of 245 nodules. These nodules were segmented in an automated fashion using a dedicated in-house workstation for lung cancer screening (CIRRUS Lung Screening, Diagnostic Image Analysis Group, Nijmegen, the Netherlands), but with the option to use one-click corrections to improve the segmentation when needed. A total of 38 nodules smaller than 6 mm were excluded from the analysis since these nodules have a high chance of being benign lesions^2,3^. An additional 37 nodules were excluded that had a *complex* structure, defined as large irregularly shaped nodules that may contain bubbles, as their appearance and texture is quite unique and not largely represented in our training data set. A similar procedure was followed by the authors of Scholten *et al*.^12^. The final test set therefore consisted of 170 subsolid nodules.

### Detection of Vessels and Solid Cores

In the first experiment, we evaluated if the proposed method is able to automatically detect vessels and solid cores within a subsolid nodule. Automatic detection of vessels and solid cores was evaluated using the 170 screen-detected nodules of the MILD study, where a connected component of two or more vessel or solid core voxels was counted as a detection. A connected component analysis (with 26 neighborhood connectivity) was performed for each class in order to verify that the method produces spatial consistent solid core and vessel detections. For each nodule the number of connected components for each class was extracted. For solid core detection, 57 nodules were found with only a single connected solid core component and 8 nodules were found with more than 1 connected solid core component (with a maximum of 4 solid core components in a single nodule). For vessel detection, 88 nodules were found with only 1 connected vascular component and 31 nodules were found with multiple connected vascular component (with a maximum of 7 vascular components in a single nodule). For ground glass, 169 nodules were found with a single connected component and only 1 nodule was found with 2 connected ground glass components.

Three observers independently inspected the set of 170 nodules and indicated if the nodules contained a vessel and/or a solid core. Inter-observer agreement was assessed for each pair of observers by computing the accuracy, sensitivity, specificity, precision, and Cohen’s kappa (*κ*)^14^. The performance of our method was evaluated by comparing the results of our method to the results of each individual observer using the same performance metrics. All results of this evaluation are presented in Table 1. For vessel detection, a substantial agreement was found between the observers (with a *κ* ranging from 0.61 to 0.67) and between our method and the observers (with a *κ* ranging from 0.60 to 0.70). For solid core detection, a moderate agreement was found between the observers (with a *κ* ranging from 0.42 to 0.59) and between our method and the observers (with a *κ* ranging from 0.34 to 0.52). Furthermore, the results reported in Table 1 show that for both vessel and solid core detection, human observers tend to be more sensitive whereas our method is more specific and precise.

**Table 1:** Evaluation of the inter-observer and method-observer agreement on the detection of vessels and solid cores in subsolid nodules.

	Kappa		Accuracy		Sensitivity		Specificity		Precision	
	vessel	solid core	vessel	solid core	vessel	solid core	vessel	solid core	vessel	solid core
*Inter-observer comparison*										
O1 vs O2	0.67 (0.56–0.78)	0.50 (0.37–0.63)	0.84	0.76	0.85	0.73	0.82	0.81	0.88	0.88
O1 vs O3	0.61 (0.48–0.73)	0.59 (0.47–0.71)	0.82	0.79	0.79	0.93	0.88	0.70	0.94	0.67
O2 vs O3	0.67 (0.55–0.78)	0.42 (0.28–0.55)	0.85	0.69	0.82	0.94	0.90	0.52	0.95	0.57
*Method-observer comparison*										
Method vs O1	0.70 (0.60–0.81)	0.41 (0.28–0.55)	0.85	0.70	0.81	0.57	0.91	0.86	0.93	0.83
Method vs O2	0.65 (0.53–0.76)	0.34 (0.21–0.48)	0.82	0.65	0.78	0.52	0.90	0.89	0.92	0.91
Method vs O3	0.60 (0.47–0.72)	0.52 (0.39–0.65)	0.80	0.77	0.72	0.69	0.98	0.82	0.99	0.72

The observers are indicated as O1 (observer 1), O2 (observer 2), and O3 (observer 3). Cohen’s *κ* values are given with 95%-confidence intervals.

### Segmentation of vessels and Solid Cores

#### Quantitative Evaluation

**Figure 2:**
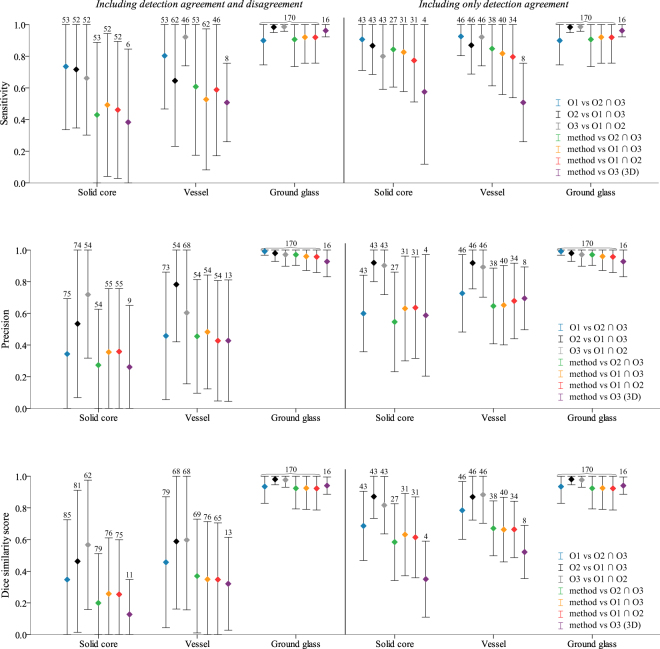
A visual representation of the inter-observer and method-observer segmentation performance for solid core, vessel, and ground glass segmentation. From top to bottom, the figures show the results in terms of sensitivity, precision, and Dice similarity score, respectively. The results are presented as the mean (indicated by the colored diamond) and the standard deviation (indicated by the whiskers). The left side of each figure shows the mean and standard deviation derived from nodules for which there was either agreement or disagreement on the presence of a class. The right side of each figure shows the mean and standard deviation derived from only the nodules for which there was agreement on the presence of a class. The number of nodules from which the means and standard deviations were calculated are reported on the top of the whiskers. It should be noted that the experiment that compares three dimensional annotations of observer 3 to the results of the method, i.e. method vs O3 (3D), was performed on a subset of 16 nodules.

A quantitative evaluation was performed to compare automatically segmented vessels and solid cores to manually drawn segmentations. Three observers manually segmented all vessel and solid core voxels on one axial slice of interest per nodule. The selected slice of interest was the axial slice with the largest cross-sectional area of the nodule. We restricted the manual segmentation process to these selected slices, since manual segmentation of all voxels in each nodule is a tedious and time-consuming task. For each pair of observers, a consensus standard was constructed using the intersection of their segmentations. To estimate the inter-observer agreement, each consensus standard was compared to the segmentations of the observer that was not considered in the consensus standard. The sensitivity, precision, and Dice similarity coefficient (*DSC*) were calculated for each nodule and all three classes, from which means and standard deviations were derived. Similarly, we evaluated the performance of our method by comparing the automatic segmentation with the segmentation of each consensus standard. As an addition to the evaluation on cross-sectional slices, a full 3D evaluation was performed by observer 3 on a subset of the data. This subset consisted of 16 size-matched nodules with a balanced amount of non-solid and part-solid nodules with and without vessels. A visual representation of all results is shown in Fig. 2 and the corresponding means and standard deviations can be found in Tables 1, 2, and 3 of the supplementary material.

**Table 2:** Qualitative scores for the proposed vessel and solid core segmentations for observer 1 (O1), observer 2 (O2), and observer 3 (O3) separately, stratified by nodule type i.e. non-solid (NS) or part-solid (PS), both with or without vessels.

	Vessel									Solid Core								
	Good			Acceptable			Unacceptable			Good			Acceptable			Unacceptable		
	O1	O2	O3	O1	O2	O3	O1	O2	O3	O1	O2	O3	O1	O2	O3	O1	O2	O3
NS, no vessels	35	21	28	0	0	0	0	0	0	32	21	25	2	0	0	1	0	3
PS, no vessels	33	44	23	0	2	0	2	0	0	14	13	7	7	8	12	14	25	4
NS, vessels	27	23	22	10	7	35	4	6	17	35	31	59	3	2	7	3	3	8
PS, vessels	42	39	24	6	16	15	11	12	6	29	18	16	17	15	16	13	34	13
*Total*	137	127	97	16	25	50	17	18	23	110	83	107	29	25	35	31	62	28

The numbers indicate the number of nodules that were scored in that specific category.

**Figure 3:**
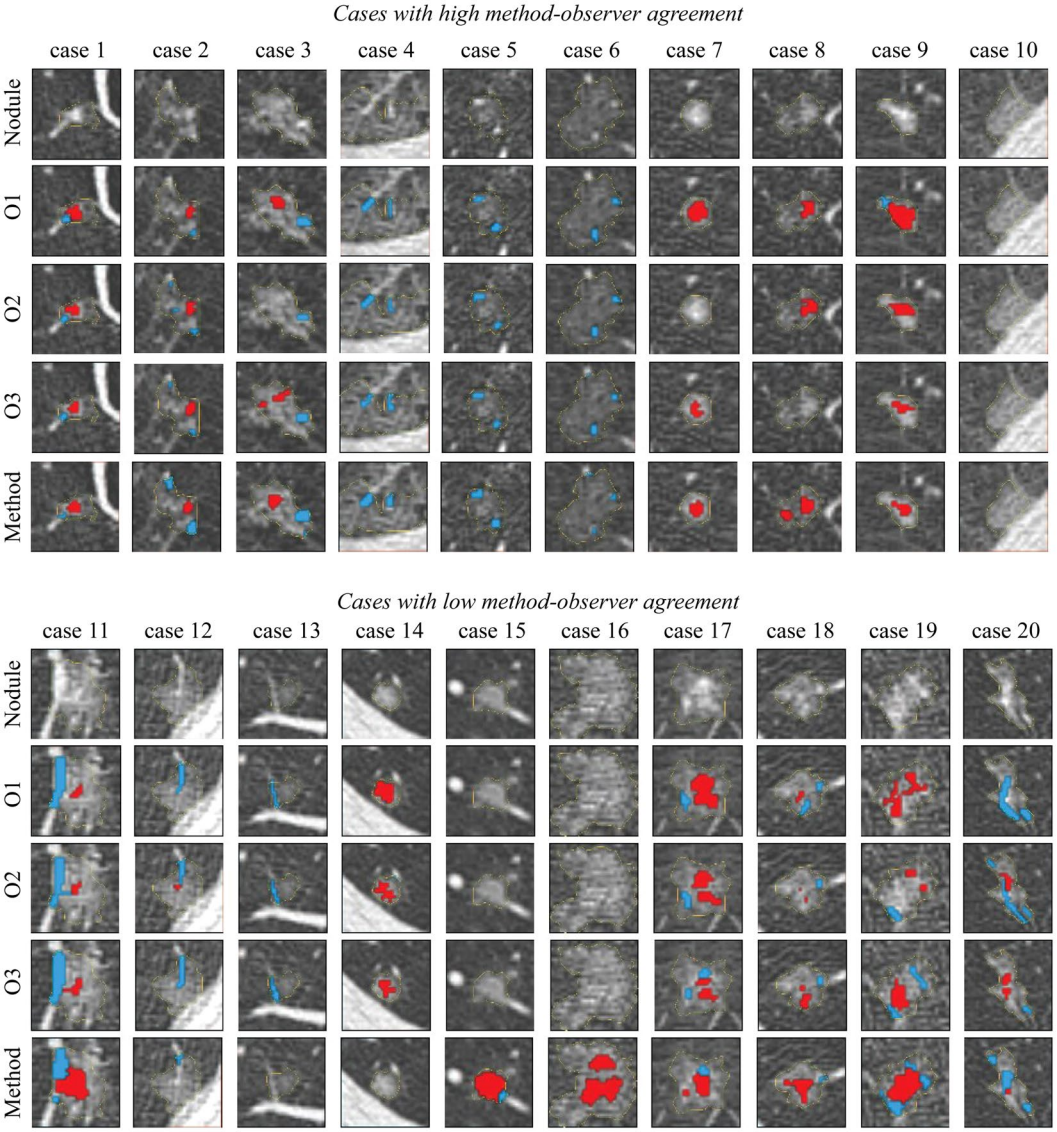
Examples of nodules with observer annotations and results of the proposed method, where the segmented vessels are indicated in blue and the segmented solid cores in red. Top: Nodules in which there is a high method-observer agreement for vessel and/or solid core segmentation. Bottom: Nodule in which there is a low method-observer agreement for vessel and/or solid core segmentation. Each column represents a separate nodule in which the first row indicates the axial slice that is considered for the evaluation. The second, third, and fourth rows show the annotations of observer 1, 2, and 3, respectively. The results of the proposed method are shown in the fifth row. All images are shown at a standard lung window level (width = 1600, center = −600) with a field of view of 10 × 10 mm.

In Fig. 2, the left side of each individual figure shows means and standard deviations that are calculated from all nodule for which we could calculate the sensitivity, precision, and *DCS*. This includes nodules for which there was either agreement or disagreement on the presence of a class. It should be noted however, that when computing an average sensitivity, precision, or *DSC* based on individual scores per nodule, the resulting number will depend on the agreement of the presence of vessels, solid cores, and GGO. For example, when the observers disagree on the presence of a class in a nodule, the sensitivity, precision, and *DSC* for that particular nodule and class will be zero. These nodules may therefore dominate the mean scores, resulting in low scores and high standard deviations. The right side of Fig. 2 therefore shows means and standard deviations calculated only from the nodules in which there is agreement on the presence of a vessel, solid core, or ground glass. The number of nodules used to calculate the means and standard deviations are reported on top of each whisker and in Tables 1, 2, and 3 of the supplementary material. It can be noticed that observers tend to be more sensitive and precise at delineating vessels and solid cores than the automatic method. This is also reflected by the higher Dice similarity coefficients for the observers. Furthermore, for both the observers and the method, vessel segmentation was done with a higher sensitivity and precision as compared to solid core segmentation. Examples of observer annotations and results of the proposed method are shown in Fig. 3.

#### Qualitative Evaluation

**Figure 4:**
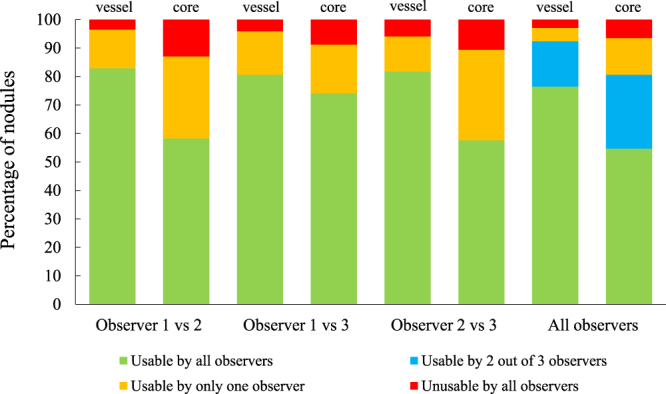
Inter-observer agreement for the usability of the proposed vessel and solid core segmentations for observer 1 and 2 (first and second bars), observer 1 and 3 (third and fourth bar), observer 2 and 3 (fifth and sixth bar), and all three observers (seventh and eight bar), where a usable segmentation is defined by a score of *good* or *acceptable*.

As a final evaluation, the automatically extract vessel and solid core segmentations of the entire nodule were presented to the three observers independently as an overlay on the original CT scan. The observers indicated for the vessels and solid cores separately if they were under-, or over-segmented and graded the quality of each segmentation as *good*, *acceptable*, or *unacceptable*. The qualitative scores given by the observers are presented in Table 2, showing that the vast majority of segmentations were scored as *good*. A segmentation was deemed usable in clinical practice if a qualitative score of *good* or *acceptable* was given. The agreement on the usability of the presented segmentations between the observers is presented in Fig. 4, showing that 92.4% of the proposed vessel segmentations and 80.6% of the proposed solid core segmentations were scored as usable by the majority of observers.

## Discussion

Mistaking a vessel for a solid core can lead to an enormous overestimation of the mass and size of the nodule and solid core, which in turn can result in an overestimated growth that can change patient management. Subsolid nodules are particularly important to identify and measure correctly since they are associated with a high malignancy probability. Our method is able to identify vessels and solid cores in subsolid nodules and may be used to provide more reliable nodule characteristics.

A substantial agreement for vessel detection was found between our method and the observers, with an average Cohen’s kappa of 0.65. A similar inter-observer agreement was found for vessel detection, indicating that the proposed automatic vessel detection performs comparable to visual detection of human observers. For the detection of a solid core, the agreement between our method and the observers was moderate, with an average Cohen’s kappa of 0.42. A slightly higher moderate agreement for solid core detection was found between the observers, showing that identification of a solid core in subsolid nodules is a more difficult and subjective task than vessel detection for both human experts and our method. These findings are not unexpected as previous studies also showed a high inter-observer variability for identifying a solid core in subsolid nodules^11,12,15,16^. In the work by Scholten *et al*.^12^, two experts independently scored 107 subsolid nodules as either being part-solid or non-solid, showing a substantial inter-observer agreement (*κ* = 0.68). This inter-observer agreement was slightly higher as compared to the inter-observer agreement for solid core detection reported in the current study. This difference in inter-observer agreement can be an effect of numerous factors, including differences in data sets, expertise of the observers, or scan parameters such as reconstruction kernels. A limitation of the study by Scholten *et al*.^12^ was that vessels were not taken into consideration in the analysis. In the method by Jacobs *et al*.^11^, a computer-aided detection (CAD) system was developed for nodule type classification. Four experts classified a set of 138 nodules as non-solid, part-solid or solid, resulting in a moderate to substantial inter-observer agreement (with *κ* values between 0.56 and 0.81) and a similar method-observer agreement (with *κ* values between 0.54 and 0.72). In the study by van Riel *et al*.^15^ a similar nodule classification task was performed on 160 pulmonary nodules by eight observers, where a moderate inter- and intra-observer agreement was reported. Although nodule classification is different from the task in the presented study, the authors of Jacobs *et al*.^11^ and van Riel *et al*.^15^ observed that the vast majority of mistakes in nodule type classification where found in differentiating part-solid from non-solid lesions. They concluded that this classification task is difficult for both human experts and CAD.

In order to validate the spatial consistency of the detected vessel and solid core segmentations, a connected component analysis was performed on each of the three output classes. This analysis showed that our method does not produce fragmented outputs in any of the classes. For example, when considering the solid core class, there were only 8 nodules in which the classifier found more than 1 connected solid core component. From these 8 nodules, the nodule with the most connected solid core components only had 4 components. These numbers are a little bit higher for vessels, which is explained by the fact that there is often more than one vessel crossing the nodule. Furthermore, the ground glass class showed to be a single connected structure in all but one nodule.

Annotating all vessels and solid cores for an entire nodule is a tedious and very time consuming task. Our quantitative evaluation by three observers was therefore performed in a single automatically selected axial slice per nodule. However, an additional full 3D evaluation was performed by one of the observers on a subset of 16 size-matched nodules with a balanced amount of non-solid and part-solid nodules with and without vessels. Although the 3D evaluation was performed on a small subset of the data, the results suggest that the mean performance is slightly lower but comparable to the mean performance derived from the the 2D evaluation. Both results show that there is a high standard deviation in all vessel and solid core segmentation measurements for both the inter-observer and method-observer agreement, and that there are relatively low precision and *DSC* scores. This indicates that correctly outlining vessels and solid cores is a difficult and subjective task. An additional effect that drives these low means and high standard deviations is the substantial amount of nodules in which the observers disagree on the presence of vessels and solid cores. Accounting for the nodules in which there was detection disagreement provides insights into the actual inter-observer and method-observer segmentation agreement. These results show that, when there is agreement on the presence of a class, the sensitivity, precision, and *DSC* scores for both the inter-observer agreement and the method-observer performance are much higher and the standard deviations are lower. Especially in terms of sensitivity, the method performs in a similar range as compared to the inter-observer agreement. However, the precision of the observers was overall higher for both vessels and solid core segmentation. This is also reflected by the higher Dice similarity coefficients for the observers. Low *DSC* scores for our method typically occurred in cases where there was also a high inter-observer variability, such as the example cases 17, 18, 19, and 20 of Fig. 3.

The obtained performance in terms of detection suggests that the proposed method could be used to identify vessels and solid cores in subsolid nodules. This could, for example, be integrated in a chest CT workstation for lung cancer screening, such as the one used in this study (CIRRUS Lung Screening, Diagnostic Image Analysis Group, Nijmegen, the Netherlands). We showed that the majority of the proposed segmentations are scored to be useful in clinical practice, which would speed up the reading procedure of subsolid nodules. However, our method showed to be less suited for precisely outlining the borders of vessels and solid cores. Therefore, the identified vessels and solid cores could be used to distinguish non-solid from part-solid nodules and to flag nodules that enclose vessels. Furthermore, the presented segmentations could trigger or initialize (semi-)automatic algorithms to refine the segmentations.

An additional analysis was performed in which three observer each scored the quality of the proposed segmentations for the entire nodule. This qualitative evaluation showed that most of the vessel and solid core segmentations were scored as usable in clinical practice by at least two of the three observers. Furthermore, only 2.9% of the vessel segmentations and 6.5% of the solid core segmentations were scored as unusable by all three observers, suggesting that our method could potentially be used to assist clinicians in assessing subsolid nodules. In the qualitative evaluation, each observer also rated the quality of the presented vessel and solid core segmentations and indicate if there was over and/or under segmentation. The main reason for scoring a segmentation as *unacceptable* was under segmentation, which was indicated to be the reason for vessels in 71% for observer 1, 83% for observer 2, and 74% for observer 3, and the reason for solid cores in 81% for observer 1, 78% for observer 2, and 46% for observer 3. The disagreement on the presence of a vessel or solid core between the observer, has a direct influence on the given qualitative scores. This means that when one observer classified a nodule as part-solid and another observer classified the same nodule as non-solid, the given quality score of that nodule can range from *good* for one observer to *unacceptable* for another. This disagreement is partly reflected by the yellow part of the bars in Fig. 4.

The extraction of three consensus standards by intersecting annotations of each pair of observer provides the opportunity to directly compare the delineation performance of human observers to that of the proposed method. However, using an intersection is at risk of making the consensus segmentation smaller which may bias the results. In order to provide a complete view of the inter-observer and method-observer performance, the sensitivity, precision, and *DSC* scores extracted from the comparisons between each individual observer (O1 vs O2, O1 vs O3, O2 vs O3), each observer and the consensus standards (O1 vs O2 ∩ O3, O2 vs O1 ∩ O3, O3 vs O1 ∩ O2), each observer and the method (method vs O1, method vs O2, method vs O3), and each consensus standard and the method (method vs O2 ∩ O3, method vs O1 ∩ O3, method vs O1 ∩ O2) are provided as supplementary material. These results show that the sensitivity, precision, and *DSC* scores are on average higher when inter-observer evaluation was performed with the consensus standard, as compared to only a single observer. This suggests that the consensus standards are not substantially smaller or less accurate than the segmentations of the individual observers.

Vascular continuity analysis was proposed in the presented method as a regularization step that smooths the transition between the vessels inside and vessels outside the nodule. This analysis aims to reduce the likelihood of voxels that were initially classified as vessel but do not match the anatomy of the vessels outside of the nodule. A potential limitation of this analysis is the dependency on a vessel segmentation outside of the nodule, as errors in this segmentation can lead to incorrect input for the regularization step. In the proposed method, we account for potential noise in the vessel segmentation outside the nodule by enforcing that a detected outer vessel has to be attached to the vascular tree. However, our method does not account for missed outer vessels, which can potentially lead to removal of correctly detected vessels inside the nodule. Nevertheless, missed vessels are typically small in diameter and will therefore only have a small effect on the mass or volume of a nodule.

The evaluation of our method was performed on a subset of subsolid nodules from the MILD study that were larger or equal to 6 mm in diameter. From this subset, 37 subsolid nodules with a *complex* structure, defined as large nodules that are irregularly shaped and may contain bubbles, were excluded before conducting the study as their appearance and texture is quite unique and not largely represented in our training data set. A similar procedure was followed by the authors of Scholten *et al*.^12^. Our method is therefore not suited to deal with these type of nodules. Especially nodules that contain bubble-like intensity are difficult to classify with the proposed three-class approach since bubble-like intensities do not belong to any of the considered classes. However, this limitation can potentially be overcome by including a substantial amount of *complex* nodules in the training procedure and extending the method to deal with bubbly-like intensities. Although improving our method to deal with these *complex* lesions is an interesting research topic, this is outside the scope of the presented paper. We further noticed that the proposed method tends to miss relative large solid cores in smaller nodules. An example of such a misclassification is shown in case 14 of Fig. 3. In this example, the solid component covers almost the entire nodule, leaving little room for areas of ground-glass. We additionally observed that smaller vessels were occasionally under-segmented or entirely missed. A possible explanation is that these kind of examples are under-represented in the training set that was used to build our classification model. However, under-segmentation of small vessels might not be clinically relevant, since they only marginally affect the volume or mass of a subsolid nodule.

In conclusion, we showed that it is feasible to automatically identify and segment vessels and solid cores in subsolid nodules. We found that from our test set of 170 subsolid nodules, 92.4% of the proposed vessel segmentations and 80.6% of the proposed solid core segmentations were labeled as usable in clinical practice by the majority of experts. We additionally showed that there is a relatively high variability in the inter-observer agreement and a low method-observer agreement in exactly outlining the borders of a vessel or solid core, which illustrates the difficulty of this task. Nevertheless, our method achieves satisfactory results in the majority of cases, making it a potential useful tool for assessing subsolid nodules in lung cancer screening and clinical routine.

## Methods

**Figure 5:**
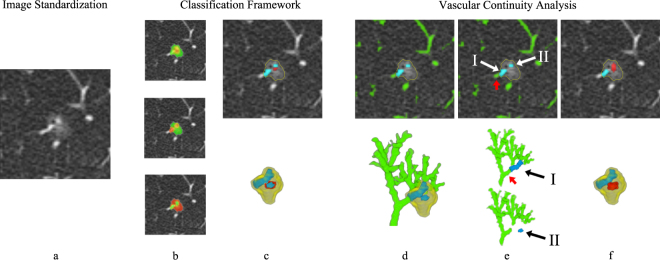
Schematic overview of the proposed method. (**a**) Axial slice of a given subsolid nodule in which an image standardization procedure is performed. (**b**) Overlay of extracted likelihoods for the solid core class (top frame), vessel class (middle frame), and ground-glass class (bottom frame), where red indicates a high and green a low likelihood. (**c**) Initial segmentation extracted from the three class likelihoods, shown in 2D (top frame) and 3D (bottom frame), where red indicates a solid core, blue indicates vessels, and the yellow outline indicates the given nodule segmentation. All unlabeled voxels within the nodule segmentation are of the ground-glass class. (**d**) A vascular continuity analysis is performed on the initial vessel segmentation inside the nodule (indicated in blue) which starts by defining the vessels outside the nodule (indicated in green), shown as a 2D overlay (top frame) or as a 3D rendering (bottom frame). (**e**) The vessels inside the nodule are divided into separate connected components (components I and II) for which vessels entering the nodules are defined. The entering vessel of I is indicated by the red arrow, whereas II is disconnected from the outside vessel and therefore has no entering vessel attached to it. These entering vessels are used to ensure an anatomically plausible continuity to the vessels inside the nodule. (**e**) Final segmentation extracted using both the initially defined likelihoods and the result from the vascular continuity analysis, shown in 2D (top frame) and 3D (bottom frame).

A schematic overview of the proposed method is shown in Fig. 5, which consists of three main parts. First, image standardization is performed on the original CT scan in order to normalize the input to our method. Next, given a nodule segmentation in 3D, a classifier is applied to classify each voxel in the segmentation as vessel (*v*), solid core (*c*), and ground-glass (*g*). For each class, the posterior probability is extracted and used to produce an initial vessel, solid core, and ground-glass segmentation. As a final step, a *vessel continuity analysis* is performed on the initial vessel segmentation in order to ensure continuity between vessels outside and inside the nodule, which is used to improve the final segmentations. These steps are described in detail in the following sections.

### Image Standardization

In order to make our method applicable to scans of different resolutions and reconstruction kernels, standardization of the input CT scan *I* was done by performing 1) resampling and 2) kernel normalization. The input CT *I* was first resampled to an isotropic resolution of 0.5 mm using linear interpolation. Since reconstruction kernels affect the spatial resolution and image noise in the reconstructed data, a kernel normalization technique^17^ was subsequently applied that transforms the resampled CT scan into an image that better matches a chosen reference reconstruction kernel. The normalization procedure decomposes a scan into several predefined frequency bands. The energy in these bands is altered to better match the average energy that is observed in a set of scans reconstructed with the chosen reference kernel. For the purpose of this study, the reference kernel was chosen to be a soft reconstruction kernel Siemens b31f.

### Classification Framework

#### Feature Extraction and Classification

A set of 3D local intensity-based voxel descriptors was extracted from the standardized scan in order to train a classifier ${\mathscr{C}}$ and extract posterior probabilities for the vessel, solid core, and ground-glass class for all voxels in a given nodule. To differentiate between the morphological shapes of vessels and solid components, multi-scale second-order spatial derivative features were extracted, i.e. the eigenvalues of the Hessian matrix (*λ*_1_, *λ*_2_*and λ*_3_, where |*λ*_1_| ≥ |*λ*_2_| ≥ |*λ*_3_|) and the gradient. All spatial derivative features were calculated for scales *σ *= 1,2 *and* 4 *mm*. Multi-scale intensity features were additionally included in order to differentiate between soft-tissue and ground glass attenuation, namely the original Hounsfield units, the Hounsfield units after Gaussian smoothing (at scales *σ* = 1,2 *and* 4 *mm*), and the standard deviation in the 26-neighborhood surrounding the sample voxel.

#### Classifier Training and Optimization Procedure

A set of 44 subsolid nodules with a diameter ranging from 6.8 to 50.6 mm (average diameter of 16.8 mm) was selected from 21 patients in order to train the classifier ${\mathscr{C}}$. The training data set consisted of low-dose CT scans with a near-isotropic resolution (average in-plane resolution was 0.71 mm ± 0.07 mm with a voxel spacing of 0.7 *mm*) and were reconstructed with a soft reconstruction kernel. Image standardization was perform on the training set prior to the training procedure. Each nodule was segmented by an experienced thoracic radiologist using a dedicated in-house workstation for lung cancer screening (CIRRUS Lung Screening, Diagnostic Image Analysis Group, Nijmegen, the Netherlands), in which the expert indicated if the nodule contained vessels and/or a solid core. This resulted in 43 nodules with vessels (37 part-solid and 6 non-solid nodule), and 1 part-solid nodule without vessels. The expert was subsequently instructed to freely annotate voxels of each of the three classes in order to collect samples to train and optimize the classifier. These annotations were made by going through the nodule in the axial view using a MeVisLab (http://www.mevislab.de) based tool developed in-house. The expert was specifically instructed to provide an heterogeneous set of training samples by annotating vessels and solid cores of multiple sizes throughout the entire nodule. A total of 38,661 vessel samples, 7,771 core samples, and 68,892 ground-glass samples were extracted from the training set of 44 nodules. The three classes were balanced by randomly selecting 7,771 samples from both the vessel and ground-glass class in order to match the number of samples in the solid core class. This random selection was done in such a way that from each nodule an approximately equal amount of samples was extracted. The final balanced training set consisted of a total of 23,313 samples.

A leave-one-patient-out cross-validation experiment was performed to select the optimal classifier for the proposed problem. Based on the set of balanced training samples, we trained several classifiers (i.e. k-Nearest Neighbor classifier^19^, Random Forest classifier^20^, Gentle Boost classifier^21^, and linear discriminant classifier) and optimized a weight *w*_*m*_ for each of the three classes (i.e. *w*_*v*_, *w*_*c*_, and *w*_*g*_) that acts as an operation point of the classifier by scaling the posterior probabilities. The optimal weights were selected by individually varying *w*_*v*_, *w*_*c*_, and *w*_*g*_ by steps of 0.01, while keeping the sum of the weights equal to 1. By multiplying these weight with the soft classification output of each classifier we extract a hard classification output which was compared to the labels of the training reference. The optimal weights were defined as the weights that minimize the differences between the hard classification and the training reference. This procedure resulted in the selection of the kNN classifier (k = 150) as the optimal classifier for this task, with the optimized weights *w*_*v*_ = 0.23, *w*_*c*_ = 0.13, and *w*_*g*_ = 0.64. Note that since the posterior probabilities were calculated in the resolution of the standardized scan, linear interpolation was used to obtain the three posterior probabilities for each voxel *x* ∈ *I* in the original resolution, i.e. ${{\mathscr{P}}}_{v}(x)$, ${{\mathscr{P}}}_{c}(x)$, and ${{\mathscr{P}}}_{g}(x)$.

#### Initial Segmentations

For the extraction of an initial vessel, solid core, and ground-glass segmentation, a set of likelihoods ${\bf{L}}(x)\in {{\mathbb{R}}}^{1\times 3}$ is defined for each *x* as ${\bf{L}}(x)=\{{w}_{m}\times {{\mathscr{P}}}_{m}(x)\}=\{{ {\mathcal L} }_{m}(x)\}$ where the index *m* = 1, 2, 3 corresponds to the vessels (v), solid cores (s), and ground-glass (g) class, respectively. Based on these likelihoods, the initial segmentations of each class ${S}_{m}^{I}$ are given by:

1 $$\begin{array}{l}{S}_{m=y}(x)=1,{\rm{where}}\,y={{\rm{argmax}}}_{m}({ {\mathcal L} }_{m}(x))\\ {S}_{m\ne y}(x)=0\end{array}$$

An example of the three likelihoods of a subsolid nodule and the resulting initial vessel segmentation ${S}_{v}^{I}(x)$, solid core segmentation ${S}_{c}^{I}(x)$, and ground-glass segmentation ${S}_{g}^{I}(x)$ is shown in Fig. 5a and b. In order to ensure smooth initial segmentations, solitary voxels in each of the three initial segmentations were replaced by the majority label of the neighboring voxels.

### Vascular Continuity Analysis

Vessels on CT appear as bright connected tubular branches that form a tree-like structure throughout the lung. The connected branches within the same vascular tree have a smooth continuation from large vessels in the central regions of the lung, to smaller vessels in the periphery of the lung. For vessels that intersect with a nodule, we can therefore assume that the part of a vessel that is inside the nodule has to be at least attached to the vessel that enters the nodule. In addition, the size (i.e. vessel diameter) of the entering vessel has to be larger or equal to the size of the attached vessel inside the nodule. By using these anatomical constrains, we define a weight *w*_*vc*_ that quantifies the smoothness of the transition between vessels entering the nodule and initially classified vessels inside the nodule. A schematic overview of this analysis is shown in Fig. 5d,e and f.

#### Defining Entering Vessels

Vascular continuity analysis was used to quantify the transition between the initially classified vessels inside the nodule ${S}_{v}^{I}$ and vessels that are outside the nodule, where the vessel segmentation outside of the nodule is given by any segmentation that produces a binary vessel segmentation. In this study we used the algorithm presented by van Dongen *et al*.^18^ to extract the vessels outside the nodule. An example of vessels inside and outside the nodule is shown in Fig. 5d, where the vessels outside are indicated in green and the vessels inside are indicated in blue.

The initial vessel segmentation ${S}_{v}^{I}$ is first divided into separate components *i*_*in*_, each connected by a 6-neighborhood as shown in Fig. 5e. For each component *i*_*in*_ the entering vessel *i*_*out*_ is defined using the following three criteria: 1) *i*_*out*_ has to be attached to *i*_*in*_, 2) *i*_*out*_ has to be larger or equal to any other outside vessel that is attached to *i*_*in*_, and 3) *i*_*out*_ has to be connected to the vascular tree. The last criterium enforces that potential noise in the vessel segmentation do not affect the vascular continuity weights. In the example in Fig. 5e, the entering vessels for the first component I is indicated by a red arrow, whereas for the second component II there is no entering vessels since the component is disconnected from the outside vessel segmentation.

#### Vascular Continuity Weight

Based on the size of the vessel inside the nodule and vessel entering the nodule, a weight *w*_*vc*_(x) is calculated for each $x\in {S}_{v}^{I}$ that quantifies the smoothness of the transition between a vessel that enters the nodule and a vessel inside the nodule. To calculate these weights, a diameter is estimated for each voxel *x* ∈ *i*_*in*_ denoted as ${d}_{in}^{i}(x)$. In addition, an average diameter is estimated from the part of the entering vessel that is close to the border of the nodule segmentation, i.e. ${d}_{out}^{i}$. Based on the ratio between ${d}_{in}^{i}(x)$ and ${d}_{out}^{i}$ a vascular continuity weight *w*_*vc*_(*x*) is defined for each $x\in {S}_{v}^{I}$ as:

2 $${w}_{vc}(x)=\{\begin{array}{cc}1, & {\rm{f}}{\rm{o}}{\rm{r}}\,{d}_{in}^{i}(x)\le {d}_{out}^{i}\\ 0, & {\rm{f}}{\rm{o}}{\rm{r}}\,{d}_{in}^{i}(x)\ge a\times {d}_{out}^{i},\\ 1-\frac{1}{(a-1)}(\frac{{d}_{in}^{i}(x)}{{d}_{out}^{i}}-1), & {\rm{o}}{\rm{t}}{\rm{h}}{\rm{e}}{\rm{r}}{\rm{w}}{\rm{i}}{\rm{s}}{\rm{e}}\end{array}$$

with *a* > 1, and where we assume that ${d}_{in}^{i}(x)\le {d}_{out}^{i}$ is a strong indication that *x* belongs to a vessels (which gives the upper boundary of the step function) and ${d}_{in}^{i}(x)\ge a\times {d}_{out}^{i}$ is a strong indication that *x* is not part of a vessel (which gives the lower boundary of the step function). The parameter *a* was empirically tuned to a value of 3 based on the training set, meaning that if the size of the vessel inside the nodule is larger than or equal to three times the entering vessel, the vascular continuity weight *w*_*vc*_(*x*) goes to zero.

#### Final Segmentation

The extracted vascular continuity weights are used to penalize the vascular likelihoods ${ {\mathcal L} }_{v}(x)$ for voxels that have an unexpected vascular anatomy. Equation 1 is used to extract the final vessel segmentation ${S}_{v}^{F}(x)$, solid core segmentation ${S}_{c}^{F}(x)$, and ground-glass segmentation ${S}_{g}^{F}(x)$, by redefining the vessel likelihood as ${ {\mathcal L} }_{v}(x)={w}_{v}\times {w}_{vc}(x)\times {{\mathscr{P}}}_{v}(x)$.

## Electronic supplementary material

Supplementary Information
