# Supplementary material for: Automatic segmentation of the solid core and enclosed vessels in subsolid pulmonary nodules
Source: Sci Rep. 2018 Jan 12;8:646. doi: 10.1038/s41598-017-19101-3 (PMC5766558; doi:10.1038/s41598-017-19101-3)
Supplement: Supplementary file 1 — Supplementary Information [file 41598_2017_19101_MOESM1_ESM.pdf]

Automatic segmentation of the solid core and enclosed vessels in subsolid pulmonary nodules

Jean-Paul Charbonnier, Kaman Chung, Ernst T. Scholten, Eva M. van Rikxoort, Colin Jacobs, Nicola Sverzellati, Mario Silva, Ugo Pastorino, Bram van Ginneken and Francesco Ciompi

1. Supplementary Table(s)

Table 1: Dice similarities scores of inter-observer and method-observer segmentation performance. This table contains each observer-observer, method-observer, observer-consensus standard, and method-consensus standard combinations. The Dice score is given for vessel segmentation, solid core segmentation and GGO segmentation separately and is presented as the mean ± standard deviation. In the first three result columns, each mean and standard deviation was derived from nodules for which there was either agreement or disagreement on the presence of a class. In the last three columns, each mean and standard deviation was derived from only the nodules for which there was agreement on the presence of a class. The number of nodules from which the means and standard deviations were calculated are reported between brackets. Note that the experiment that compares three dimensional annotations of observer 3 to the results of the method, i.e. method vs O3 (3D), was performed on a subset of 16 nodules.

	Dice (*all nodules*)			Dice (*detection agreement nodules*)		
	solid core	vessel	GGO	solid core	vessel	GGO
O1 vs O2 ⋂ O3	0.35 ± 0.38 (85)	0.46 ± 0.41 (79)	0.93 ± 0.11 (170)	0.69 ± 0.22 (43)	0.78 ± 0.18 (46)	0.93 ± 0.11 (170)
O2 vs O1 ⋂ O3	0.46 ± 0.45 (81)	0.59 ± 0.43 (68)	0.98 ± 0.03 (170)	0.87 ± 0.14 (43)	0.87 ± 0.15 (46)	0.98 ± 0.03 (170)
O3 vs O1 ⋂ O2	0.57 ± 0.41 (62)	0.60 ± 0.44 (68)	0.98 ± 0.05 (170)	0.82 ± 0.18 (43)	0.88 ± 0.18 (46)	0.98 ± 0.05 (170)
O1 vs O2	0.33 ± 0.37 (105)	0.38 ± 0.38 (87)	0.92 ± 0.11 (170)	0.67 ± 0.21 (52)	0.72 ± 0.18 (46)	0.92 ± 0.11 (170)
O1 vs O3	0.36 ± 0.34 (89)	0.46 ± 0.39 (97)	0.91 ± 0.11 (170)	0.61 ± 0.21 (52)	0.72 ± 0.21 (62)	0.91 ± 0.11 (170)
O2 vs O3	0.44 ± 0.39 (89)	0.49 ± 0.42 (85)	0.96 ± 0.05 (170)	0.74 ± 0.19 (53)	0.78 ± 0.21 (53)	0.96 ± 0.05 (170)
Method vs O2 ⋂ O3	0.20 ± 0.31 (79)	0.37 ± 0.36 (69)	0.92 ± 0.13 (170)	0.58 ± 0.24 (27)	0.67 ± 0.17 (38)	0.92 ± 0.13 (170)
Method vs O1 ⋂ O3	0.26 ± 0.35 (76)	0.35 ± 0.36 (76)	0.92 ± 0.13 (170)	0.63 ± 0.26 (31)	0.66 ± 0.20 (40)	0.92 ± 0.13 (170)
Method vs O1 ⋂ O2	0.25 ± 0.35 (75)	0.35 ± 0.36 (65)	0.92 ± 0.14 (170)	0.61 ± 0.26 (31)	0.66 ± 0.18 (34)	0.92 ± 0.14 (170)
Method vs O1	0.26 ± 0.31 (90)	0.26 ± 0.32 (92)	0.87 ± 0.16 (170)	0.57 ± 0.18 (41)	0.60 ± 0.18 (40)	0.87 ± 0.16 (170)
Method vs O2	0.19 ± 0.28 (99)	0.33 ± 0.33 (72)	0.91 ± 0.13 (170)	0.53 ± 0.21 (36)	0.60 ± 0.17 (39)	0.91 ± 0.13 (170)
Method vs O3	0.22 ± 0.31 (85)	0.32 ± 0.34 (91)	0.91 ± 0.14 (170)	0.55 ± 0.23 (34)	0.63 ± 0.19 (46)	0.91 ± 0.14 (170)
Method vs O3 (3D)	0.13 ± 0.22 (11)	0.32 ± 0.29 (13)	0.94 ± 0.05 (16)	0.35 ± 0.24 (4)	0.52 ± 0.17 (8)	0.94 ± 0.05 (16)

Table 2: Sensitivity of inter-observer and method-observer segmentation performance. This table contains each observer-observer, method-observer, observer-consensus standard, and method-consensus standard combinations. The sensitivity is given for vessel segmentation, solid core segmentation and GGO segmentation separately and is presented as the mean ± standard deviation. In the first three result columns, each mean and standard deviation was derived from nodules for which there was either agreement or disagreement on the presence of a class. In the last three columns, each mean and standard deviation was derived from only the nodules for which there was agreement on the presence of a class. The number of nodules from which the means and standard deviations were calculated are reported between brackets. Note that the experiment that compares three dimensional annotations of observer 3 to the results of the method, i.e. method vs O3 (3D), was performed on a subset of 16 nodules.

	Sensitivity (*all nodules*)			Sensitivity (*detection agreement nodules*)		
	solid core	vessel	GGO	solid core	vessel	GGO
O1 vs O2 $\bigcap$ O3	0.74 ± 0.40 (53)	0.80 ± 0.34 (53)	0.90 ± 0.15 (170)	0.91 ± 0.20 (43)	0.93 ± 0.12 (46)	0.90 ± 0.15 (170)
O2 vs O1 $\bigcap$ O3	0.72 ± 0.37 (52)	0.65 ± 0.41 (62)	0.98 ± 0.03 (170)	0.87 ± 0.18 (43)	0.87 ± 0.18 (46)	0.98 ± 0.03 (170)
O3 vs O1 $\bigcap$ O2	0.66 ± 0.36 (52)	0.92 ± 0.18 (46)	0.99 ± 0.03 (170)	0.80 ± 0.21 (43)	0.92 ± 0.18 (46)	0.99 ± 0.03 (170)
O1 vs O2	0.40 ± 0.34 (77)	0.39 ± 0.37 (78)	0.98 ± 0.05 (170)	0.60 ± 0.23 (52)	0.65 ± 0.23 (46)	0.98 ± 0.05 (170)
O1 vs O3	0.36 ± 0.32 (77)	0.54 ± 0.36 (78)	0.98 ± 0.04 (170)	0.53 ± 0.25 (52)	0.68 ± 0.27 (62)	0.98 ± 0.04 (170)
O2 vs O3	0.49 ± 0.40 (82)	0.80 ± 0.30 (57)	0.97 ± 0.05 (170)	0.75 ± 0.21 (53)	0.86 ± 0.21 (53)	0.97 ± 0.05 (170)
Method vs O2 $\bigcap$ O3	0.43 ± 0.46 (53)	0.61 ± 0.43 (53)	0.91 ± 0.17 (170)	0.84 ± 0.24 (27)	0.85 ± 0.23 (38)	0.91 ± 0.17 (170)
Method vs O1 $\bigcap$ O3	0.49 ± 0.45 (52)	0.53 ± 0.45 (62)	0.92 ± 0.16 (170)	0.83 ± 0.25 (31)	0.82 ± 0.26 (40)	0.92 ± 0.16 (170)
Method vs O1 $\bigcap$ O2	0.46 ± 0.43 (52)	0.59 ± 0.42 (46)	0.92 ± 0.16 (170)	0.77 ± 0.26 (31)	0.80 ± 0.26 (34)	0.92 ± 0.16 (170)
Method vs O1	0.35 ± 0.37 (77)	0.34 ± 0.40 (78)	0.91 ± 0.17 (170)	0.65 ± 0.24 (41)	0.67 ± 0.30 (40)	0.91 ± 0.17 (170)
Method vs O2	0.32 ± 0.40 (82)	0.54 ± 0.42 (57)	0.90 ± 0.18 (170)	0.72 ± 0.26 (36)	0.79 ± 0.25 (39)	0.90 ± 0.18 (170)
Method vs O3	0.42 ± 0.44 (64)	0.45 ± 0.44 (82)	0.90 ± 0.18 (170)	0.79 ± 0.26 (34)	0.80 ± 0.25 (46)	0.90 ± 0.18 (170)
Method vs O3 (3D)	0.38 ± 0.46 (6)	0.51 ± 0.25 (8)	0.96 ± 0.04 (16)	0.58 ± 0.46 (4)	0.51 ± 0.25 (8)	0.96 ± 0.04 (16)

Table 3: Precision of inter-observer and method-observer segmentation performance. This table contains each observer-observer, method-observer, observer-consensus standard, and method-consensus standard combinations. The precision is given for vessel segmentation, solid core segmentation and GGO segmentation separately and is presented as the mean ± standard deviation. In the first three result columns, each mean and standard deviation was derived from nodules for which there was either agreement or disagreement on the presence of a class. In the last three columns, each mean and standard deviation was derived from only the nodules for which there was agreement on the presence of a class. The number of nodules from which the means and standard deviations were calculated are reported between brackets. Note that the experiment that compares three dimensional annotations of observer 3 to the results of the method, i.e. method vs O3 (3D), was performed on a subset of 16 nodules.

	Precision (*all nodules*)			Precision (*detection agreement nodules*)		
	solid core	vessel	GGO	solid core	vessel	GGO
O1 vs O2 ⋂ O3	0.34 ± 0.35 (75)	0.46 ± 0.40 (73)	0.99 ± 0.02 (170)	0.60 ± 0.24 (43)	0.73 ± 0.24 (46)	0.99 ± 0.02 (170)
O2 vs O1 ⋂ O3	0.53 ± 0.47 (74)	0.78 ± 0.36 (54)	0.98 ± 0.05 (170)	0.92 ± 0.12 (43)	0.92 ± 0.16 (46)	0.98 ± 0.05 (170)
O3 vs O1 ⋂ O2	0.72 ± 0.40 (54)	0.60 ± 0.45 (68)	0.97 ± 0.07 (170)	0.90 ± 0.18 (43)	0.89 ± 0.19 (46)	0.97 ± 0.07 (170)
O1 vs O2	0.55 ± 0.45 (82)	0.71 ± 0.38 (57)	0.89 ± 0.16 (170)	0.86 ± 0.19 (52)	0.88 ± 0.16 (46)	0.89 ± 0.16 (170)
O1 vs O3	0.70 ± 0.38 (64)	0.65 ± 0.40 (82)	0.88 ± 0.17 (170)	0.86 ± 0.20 (52)	0.86 ± 0.18 (62)	0.88 ± 0.17 (170)
O2 vs O3	0.67 ± 0.37 (64)	0.51 ± 0.42 (82)	0.96 ± 0.08 (170)	0.81 ± 0.23 (53)	0.78 ± 0.24 (53)	0.96 ± 0.08 (170)
Method vs O2 ⋂ O3	0.27 ± 0.35 (54)	0.46 ± 0.36 (54)	0.97 ± 0.07 (170)	0.55 ± 0.31 (27)	0.65 ± 0.24 (38)	0.97 ± 0.07 (170)
Method vs O1 ⋂ O3	0.36 ± 0.40 (55)	0.48 ± 0.36 (54)	0.96 ± 0.09 (170)	0.63 ± 0.33 (31)	0.65 ± 0.25 (40)	0.96 ± 0.09 (170)
Method vs O1 ⋂ O2	0.36 ± 0.40 (55)	0.43 ± 0.38 (54)	0.96 ± 0.10 (170)	0.64 ± 0.32 (31)	0.68 ± 0.24 (34)	0.96 ± 0.10 (170)
Method vs O1	0.46 ± 0.36 (56)	0.47 ± 0.35 (56)	0.87 ± 0.17 (170)	0.63 ± 0.25 (41)	0.66 ± 0.22 (40)	0.87 ± 0.17 (170)
Method vs O2	0.33 ± 0.33 (56)	0.42 ± 0.35 (56)	0.95 ± 0.09 (170)	0.51 ± 0.28 (36)	0.60 ± 0.26 (39)	0.95 ± 0.09 (170)
Method vs O3	0.32 ± 0.34 (56)	0.50 ± 0.33 (56)	0.96 ± 0.08 (170)	0.52 ± 0.29 (34)	0.61 ± 0.25 (46)	0.96 ± 0.08 (170)
Method vs O3 (3D)	0.26 ± 0.39 (9)	0.43 ± 0.38 (13)	0.93 ± 0.10 (16)	0.59 ± 0.38 (4)	0.70 ± 0.20 (8)	0.93 ± 0.10 (16)
